# Supplementary material for: Pathogen infection induces sickness behaviors through neuromodulators linked to stress and satiety in C. elegans
Source: Nat Commun. 2025 Apr 3;16:3200. doi: 10.1038/s41467-025-58478-y (PMC11968842; doi:10.1038/s41467-025-58478-y)
Supplement: Supplementary file 1 — Supplementary Information [file 41467_2025_58478_MOESM1_ESM.pdf]

SUPPLEMENTARY INFORMATION

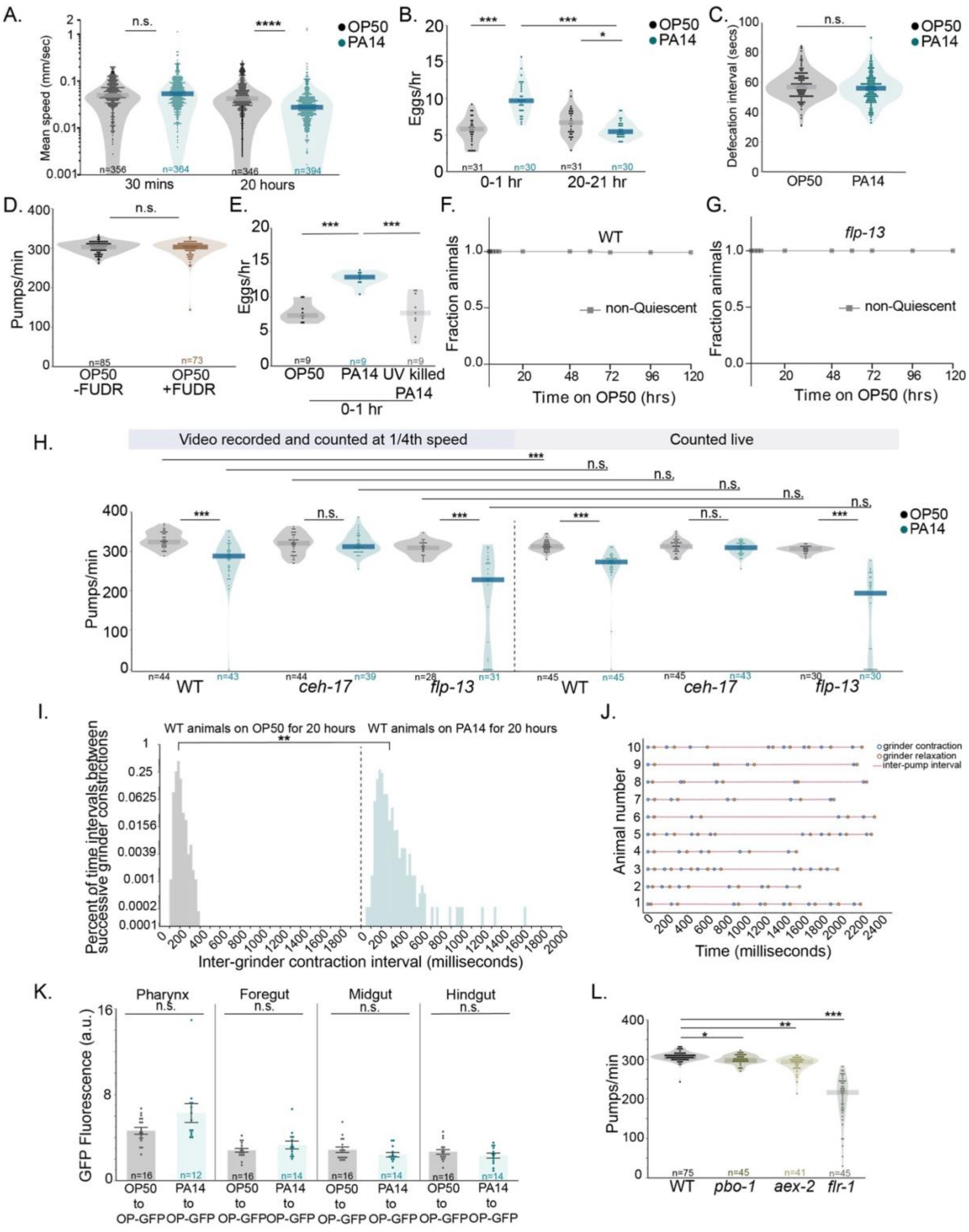

Figure S1, Related to Figure 1

(A) Mean speed (mm/sec) of wild-type animals exposed to indicated bacterial strains for 30 mins and 20 hours; Y axis is on a log scale. These animals, as well as all others in the study, were grown on *E. coli* OP50 until they were one day old adults. They were then transferred to plates containing the indicated bacteria and time point of the transfer was considered t=0. Dots indicate individual animals; dark bar indicates mean. n= 346-396 animals per condition; ****$p<0.0001$, n.s. indicates not significant, with Wilcoxon rank sum test.

(B) Egg-laying rate of wild-type animals exposed to indicated bacteria for 1 hour right after transfer to said bacteria or for 1 hour after 20 hours of exposure to bacteria. Each data point indicates eggs laid per animal per hour per plate; dark bar indicates mean. n=30-31 plates, with each plate containing 10 animals; *$p<0.01$, ****$p<0.0001$, n.s. indicates not significant, with Wilcoxon rank sum test.

(C) Defecation rate represented as inter-DMP (defecation motor program) interval for wild-type animals exposed to indicated bacteria for 20 hours. Dots indicate individual animals; dark bar indicates mean. n=113-125 animals; n.s. indicates not significant ($p>0.05$) with Wilcoxon rank sum test.

(D) Feeding rates of day-matched WT animals after overnight incubation on plates seeded with OP50, treated with or without 0.1mg/ml floxuridine (FUDR). FUDR-treated animals were selected for the expected no egg production phenotype and were tested for pharyngeal pumping rates after being placed on OP50 plates without FUDR for 1 hour. n= 73-85; n.s. indicates not significant ($p>0.05$), tested with Wilcoxon rank sum test.

(E) Egg-laying rate of wild-type animals exposed to indicated bacteria for 1 hour in absence of any prior exposure to PA14. Each data point indicates eggs laid per animal per hour; dark bar indicates mean. n=9 plates for each condition, with each plate containing 10 animals; ***$p<0.001$, with Wilcoxon rank sum test.

(F-G) Time course analysis of how animal behavior changes on OP50 bacterial controls for Fig 1A and 1J. Time indicated on the x-axis is the number of hours since animals were first placed on OP50 bacterial lawns as one-day old adults. The indicated line plots depict the fraction of animals that exhibited the indicated phenotypes at that time. Each data point indicates the mean fraction of the indicated phenotype, and surrounding error bars indicate the standard deviation. For each genotype, 10 animals were placed on a single plate; two replicates with three plates each were performed. No quiescence or death was observed when WT or *flp-13* mutants were placed on OP50, as indicated by 100% non-quiescence throughout the time course. This is in contrast to their phenotypes in Fig 1A and 1J, respectively.

(H) Feeding rates of WT, *flp-13*, and *ceh-17* animals measured consecutively with manual counting methods and video recording after 20 hours of exposure to OP50 and PA14. Recorded videos were slowed down to ¼th speed and number of pumps were tallied during this slow playback (see Methods). n=30-45 animals per condition. Wilcoxon rank sum test was performed to test between OP50 vs PA14 for each strain. ***$p<0.001$, n.s. indicates not significant ($p>0.05$). In addition, we performed Wilcoxon rank sum test with Bonferroni correction to

examine the difference between the two counting methods for each of the six experimental conditions.

(I) Distribution of inter-pump-intervals for the two experimental groups indicated. Y axis is on a log scale. Video recordings were time stamped for grinder contractions at $1/4^{th}$ speed and the interval between consecutive grinder contractions was quantified. n=43-44 animals with ~4000 time intervals for each condition. Wilcoxon rank sum test was run on randomly subsampled datasets (determined by power analysis) to compare between the two distributions, **p<0.01.

(J) Behavioral traces from 10 WT animals on PA14 for 20 hours, depicting times of grinder contraction and relaxation. A subset of long time intervals between grinder contractions for WT animals on PA14 for 20 hours were re-examined at $1/8^{th}$ speed and time stamped for both grinder contraction and relaxation. Time between consecutive grinder movements were plotted for these video segments. Each row represents a different animal. See Methods for a note about the bounds of precision of these measurements.

(K) Quantification of fluorescent intensities of GFP-tagged OP50 ingested by WT animals for 1 hour after 20 hours of exposure to non-fluorescent OP50 or PA14 bacteria. n=13-16 animals per genotype per bacterial condition; n.s. indicates not significant (p>0.05) with Wilcoxon rank sum test, with Bonferroni multiple hypothesis correction.

(L) Feeding rates of day-matched WT and mutant strains after 20 hours of incubation on OP50 bacteria. N= 45 animals per strain, *p<0.05, **p<0.01, ***p<0.001 with Wilcoxon rank sum test, with multiple hypothesis correction.

Source data are provided as a source data file.

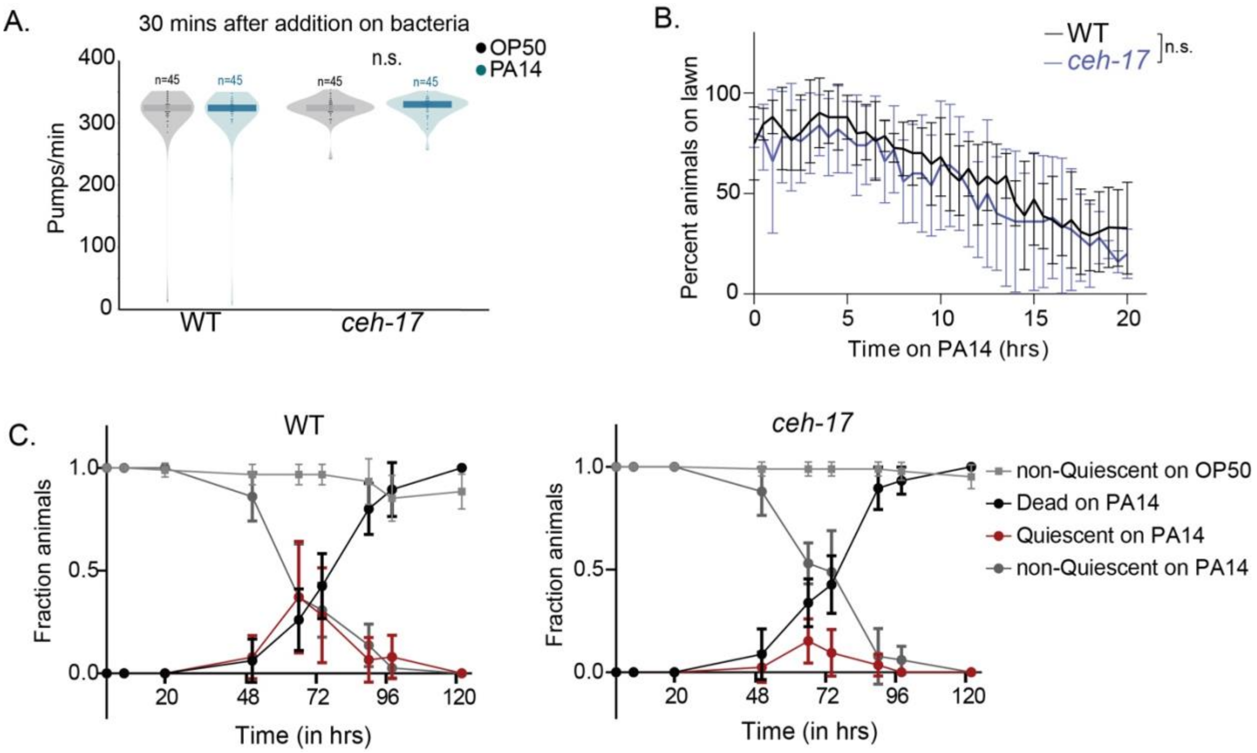

Figure S2, Related to Figure 1

(A) Feeding rates of animals on indicated bacteria upon initial exposure. Animals were left on the plate for 30 minutes for acclimatization before testing. Ns are individual animals. n.s. indicates not significantly different as tested with Wilcoxon rank-sum tests.

(B) Lawn leaving rates of wild-type vs *ceh-17* animals on PA14 over 20 hrs. For each genotype, animals were placed on a lawn of PA14, and the percent animals on the lawn were quantified over time (n=5-6 replicates with 10 animals each for each genotype). No significant difference observed between lawn leaving rates based on two-way ANOVA.

(C) Time course analysis of how behavior and viability changes over the course of infection in *ceh-17* (left) and WT (right) animals. The time indicated on the x-axis is the number of hours since animals were first placed on OP50 or PA14 bacterial lawns as one day old adults. The indicated line plots depict the fraction of animals that exhibited the indicated phenotypes at that time point. Data are shown for wild-type and *ceh-17(np1)* animals. For each genotype, 10 animals were placed on a single plate; two to three replicates with three plates each were tested. Median time point of quiescence observations was not significantly different between PA14-infected WT and *ceh-17* animals n.s. Despite no change in the onset of quiescence or death, peak fraction quiescence was significantly higher in WT animals compared to *ceh-17* mutants (0.39 vs 0.2), **p<0.01. Median time point for 50% death was not significantly different between PA14-infected WT and *ceh-17* animals, n.s. Median values compared via two tailed Mann-Whitney test.

Source data are provided as a source data file.

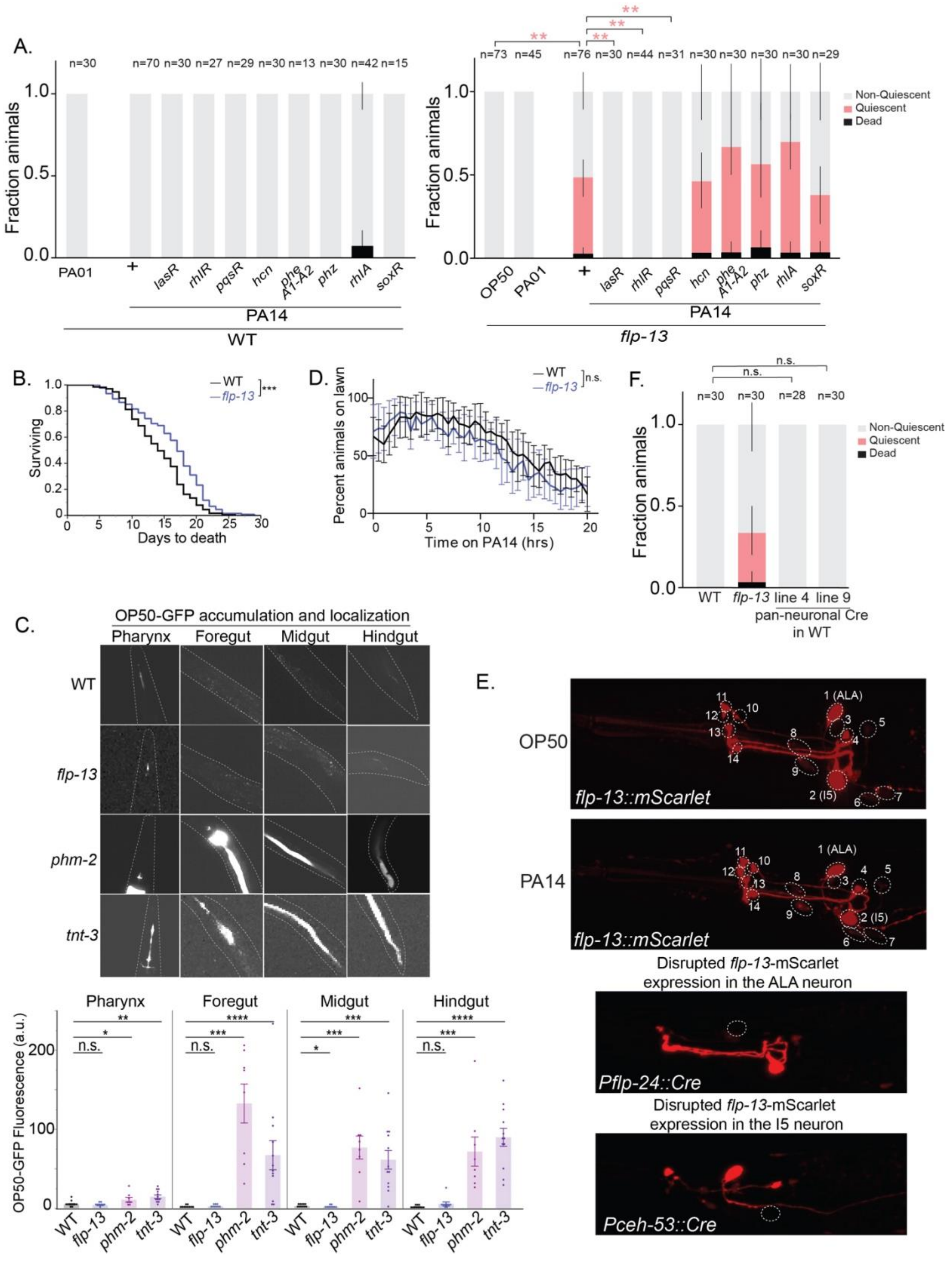

Figure S3, Related to Figure 2

(A) Quiescence and death in animals of the indicated genotypes, shown as fraction of the total population of assayed animals after 20 hours of exposure on indicated bacterial mutants. Error bars represent the 95% confidence interval for the bootstrapped mean fraction values of animals that were quiescent, non-quiescence, and dead, respectively. ** $p < 0.01$, fraction quiescence compared via Chi-square test with Bonferroni correction.

(B) Kaplan Meier curve indicating lifespans of WT and *flp-13* animals on OP50. Three independent replicates with 2-3 plates per replicate per strain (each plate containing a population of 30 animals) were run with *daf-2* controls run in parallel. The experiment was stopped when the last of the WT or *flp-13* animals died; 65.3% of the *daf-2* animals were alive at the endpoint of the experiment. Log-rank test indicates that WT and *flp-13* lifespans are significantly different, *** $p<0.001$ with median time to death being 14 days for WT and 17 days for *flp-13* animals.

(C) (Top) Representative images of accumulation of GFP-tagged OP50 in head region (pharynx), foregut, midgut, and hindgut regions of animals after being fed on GFP-tagged OP50 for 20 hours. Dotted lines indicate outlines of animals. (Bottom) Quantification of GFP intensity in the pharynx, foregut, midgut, and hindgut regions (a.u. indicates arbitrary fluorescence units). *phm-2* and *tnt-3* are positive controls mutants known to be defective in grinding bacteria. n=8-12 animals per strain; * $p<0.05$, ** $p<0.01$, *** $p<0.001$, **** $p<0.0001$, n.s. indicates not significant, with Wilcoxon rank sum test.

(D) Lawn leaving rates of wild-type vs *flp-13* animals on PA14 over 20 hrs. For each genotype, animals were placed on a lawn of PA14, and the percent animals on the lawn were quantified over time (n=9 replicates with 10 animals in each replicate for each genotype). n.s. indicates no significant interaction based on two-way ANOVA.

(E) (Top) Fluorescence images of *flp-13(syb6180 syb6395)* animals, after exposure to either OP50 or PA14 for 20 hours, beginning at the first day of adulthood. Images are shown for the head region. No fluorescence was observed in the ventral cord or tail. Dotted white circles indicate distinct neurons, some of which have been identified. In addition, no non-neuronal expression of mScarlet was detected. (Bottom) Fluorescence images of *flp-13(syb6180 syb6395)* animals expressing *Pflp-24::Cre* (left) and *Pceh-53::Cre* (right) indicating absence of mScarlet fluorescence in ALA and I5 respectively. The top panel is overexposed to be able to visualize all potential sites of *flp-13* expression.

(F) Quiescence and death in animals of the indicated genotypes after 20 hours of infection on PA14, shown as fraction of the total population of assayed animals on indicated strains. Error bars represent the 95% confidence interval for the bootstrapped mean fraction values of animals that were quiescent, non-quiescence, and dead, respectively. n.s. indicates not significantly different for fraction quiescence compared via Chi-square test with Bonferroni correction.

Source data are provided as a source data file.

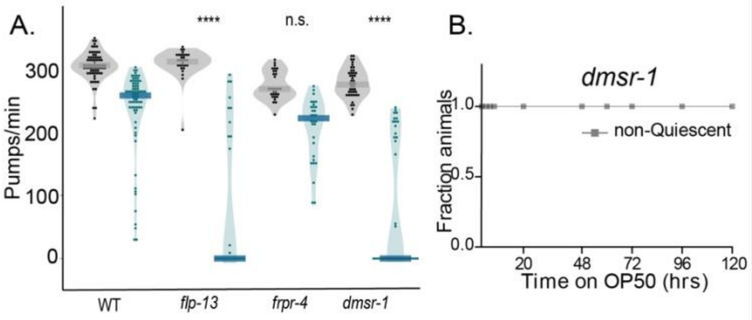

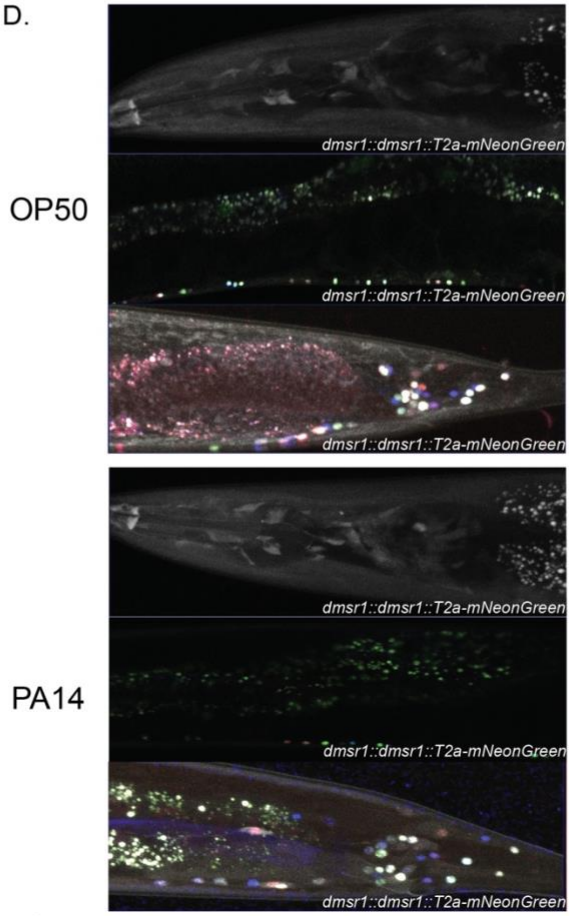

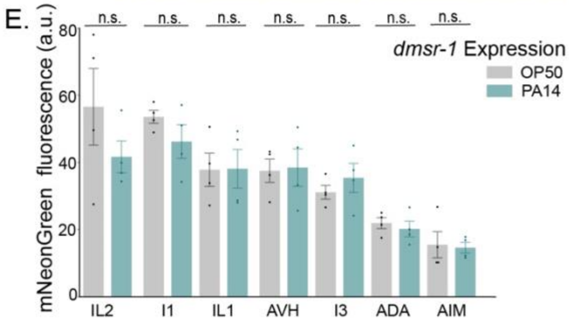

C.

LIST OF NEURONS OBSERVED	OP50	PA14
ADA	4/4	4/4
AIM	4/4	4/4
AIN	4/4	4/4
AIZ	4/4	4/4
ALN	3/3	3/3
ASI	4/4	4/4
ASJ	4/4	4/4
ASK	4/4	4/4
AVD	4/4	4/4
AVE	4/4	4/4
AVH	4/4	4/4
AVJ	4/4	4/4
BAG	4/4	4/4
CEP_DV	4/4	4/4
DA9	4/4	4/4
I1	4/4	4/4
I3	4/4	4/4
I4	4/4	4/4
IL1	4/4	4/4
IL2_DV	4/4	4/4
LUA	4/4	4/4
M1	4/4	4/4
M3	4/4	4/4
MC	4/4	4/4
OLQ_DV	4/4	4/4
PDA	3/3	3/3
PDB	3/3	3/3
PLN	3/3	3/3
PQR	4/4	4/4
PVC	3/3	3/3
PVW	4/4	4/4
RIC	4/4	4/4
RID	4/4	4/4
RIH	4/4	4/4
RIP	4/4	4/4
RMD_DVLR	4/4	4/4
RME_DV	4/4	4/4
SIB	4/4	4/4
SMD	4/4	4/4
URA	4/4	4/4
URB	4/4	4/4
URX	4/4	4/4
VA12	4/4	4/4

Figure S4, Related to Figure 3.

(A) Feeding rates in response to 20 hours of PA14 infection, in relation to the quiescence phenotypes observed in the same animals as reported in Fig 3A. Asterisks above each mutant indicate whether the PA14-induced feeding decrease for that mutant was significantly different from the decrease observed in day matched wild-type animals. ****$p<0.0001$ represents empirical P-value with Bonferroni correction. Multiple mutants were tested alongside the same control wild-type animals on a given experimental day. n.s. indicates not significantly different.

(B) Time course analysis of how animal behavior changes on OP50 bacterial controls for Fig 1A and 1J. Time indicated on the x-axis is the number of hours since animals were first placed on OP50 bacterial lawns as one day old adults. The indicated line plots depict the fraction of animals that exhibited the indicated phenotypes at that time point. Each data point indicates mean fraction of the indicated phenotype, and surrounding error bars indicate standard deviation. For each genotype, 10 animals were placed on a single plate; two replicates with three plates each were performed. No quiescence or death was observed when *dmsr*-1 mutants were placed on OP50, as indicated by 100% non-quiescence throughout the time course. This is in contrast to its phenotype in Fig 3C.

(C) List of neurons expressing *dmsr-1* identified in animals exposed to OP50 or PA14 for 20 hours. Each row depicts a neuron class, columns indicate bacterial conditions with number of times said neuron was observed over total number of observations.

(D) Representative fluorescent images on OP50 (top) and PA14 (bottom) after 20 hours. Top panel for each bacterial condition indicates the expression of *dmsr-1* tagged with mNeonGreen without the overlay of the NeuroPAL transgene (refer Fig 3D for image with NeuroPAL overlay). Middle panel indicates the mid-body of the worm, and bottom panel indicates the tail region for each bacterial condition with an overlay of NeuroPAL.

(E) Expression levels of a subset of neurons showing highest expression of *dmsr-1* across the head region of the animal in infected (PA14) and uninfected (OP50) contexts (as tabulated in Fig S3C). For some neurons with L/R/D/V pairs, the brighter cell (closer to the objective) was chosen. n.s. indicates no difference ($p>0.05$) in *dmsr-1* expression was found for that neuron class upon infection, as tested by Wilcoxon rank sum test.

Source data are provided as a source data file.

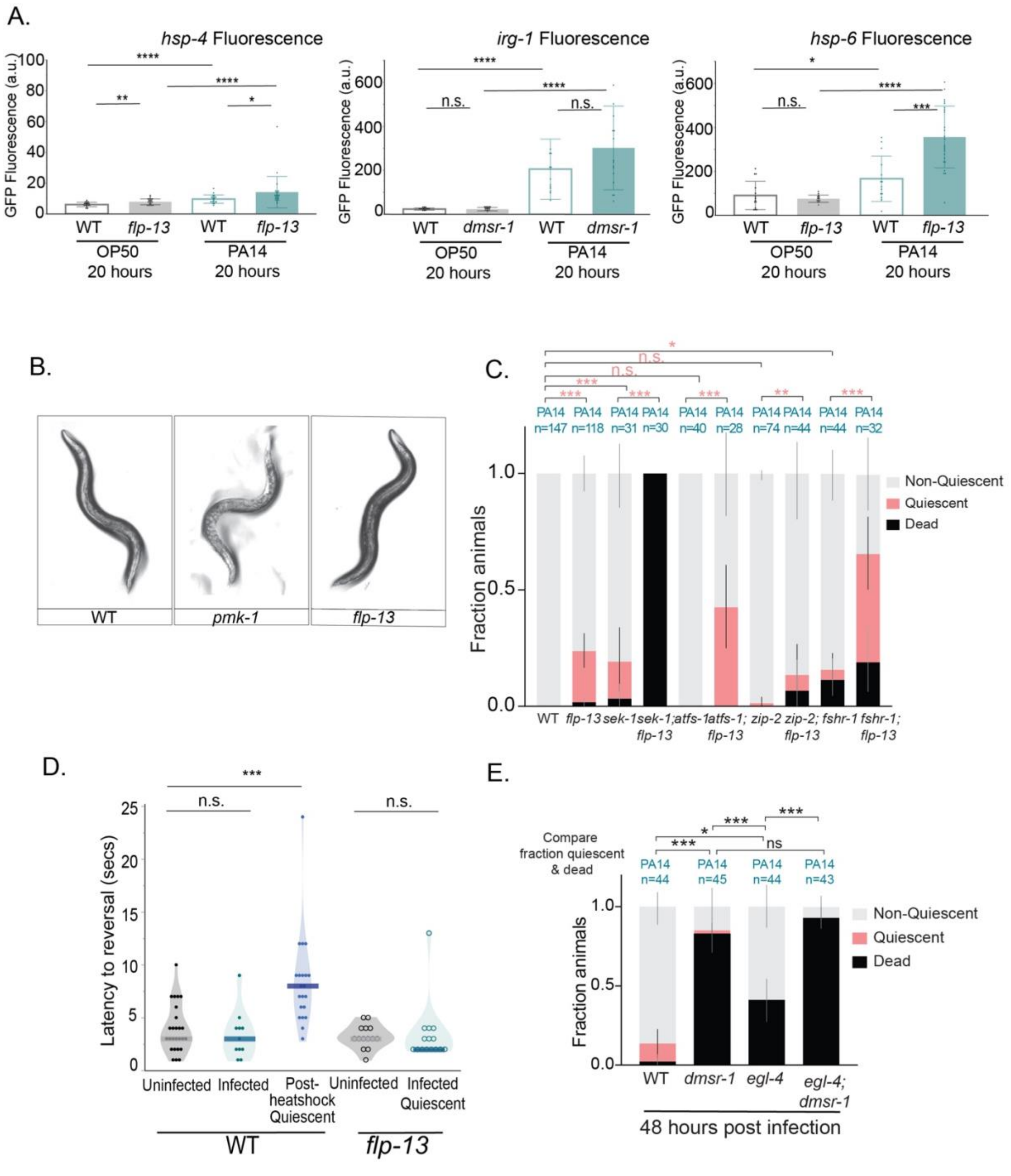

Figure S5, Related to Figure 3

(A) Quantification of fluorescent intensities of GFP tagged *hsp-4*, *irg-1* and *hsp-6* in WT and *flp-13* or *dmsr-1* animals 20 hours after being placed on indicated bacteria. n = 16-23 (for *hsp-4::GFP),* 13-14 (for *irg-1::GFP*), 13-24 (for *hsp-6::GFP)* animals per genotype per bacterial condition;. *p<0.05, ** p<0.01, ***p<0.001, ****p<0.0001, n.s. indicates not significant (p>0.05) with Wilcoxon rank sum test.

(B) Representative images of wild-type, immune pathway defective mutant *pmk-1*, and *flp-13* animals after 20 hours of exposure to PA14. Unlike *pmk-1* animals which look visibly sick, *flp-13* animals look similar to wild-types.

(C) PA14-induced quiescence and death in animals of the indicated genotypes, shown as fraction of the total population of assayed animals. Alleles used include *sek-1(km4), sek-1(km4);flp-13(tm2427), atfs-1(null), atfs-1(null);flp-13(tm2427); zip-2(ok3730), zip-2(ok3730);flp-13(tm2427), fshr-1(ok778), and fshr-1(ok778);flp-13(tm2427)*. Error bars represent the 95% confidence interval for the bootstrapped mean fraction values of animals that were quiescent, non-quiescent, and dead, respectively. *p<0.05, **p<0.01,***p<0.0001, fraction quiescent compared via Chi-square test with Bonferroni correction.

(D) Latency to arousal measured by exposing animals to blue light which leads to a robust aversive response with onset of reversals in wild-type uninfected animals, wild-type animals post heat shock and quiescent *flp-13* animals post PA14 infection. n=14-26 animals per genotype per condition; ***p<0.001, n.s. indicates not significantly different (p>0.05) with Wilcoxon rank sum test.

(E) PA14-induced quiescence and death after 48 hours (2 days) of infection in animals of the indicated genotypes, shown as fraction of the total population of assayed animals. Data after 20 hours of infection are shown in Fig. 3J. Alleles used are *egl-4(n478)* and *dmsr-1(qn45)*. Error bars represent the 95% confidence interval for the bootstrapped mean fraction values of animals that were quiescent, non-quiescent, and dead, respectively. ***p< 0.001, *p<0.01 fraction quiescent and dead compared via Chi-square test with Bonferroni correction.

Source data are provided as a source data file.

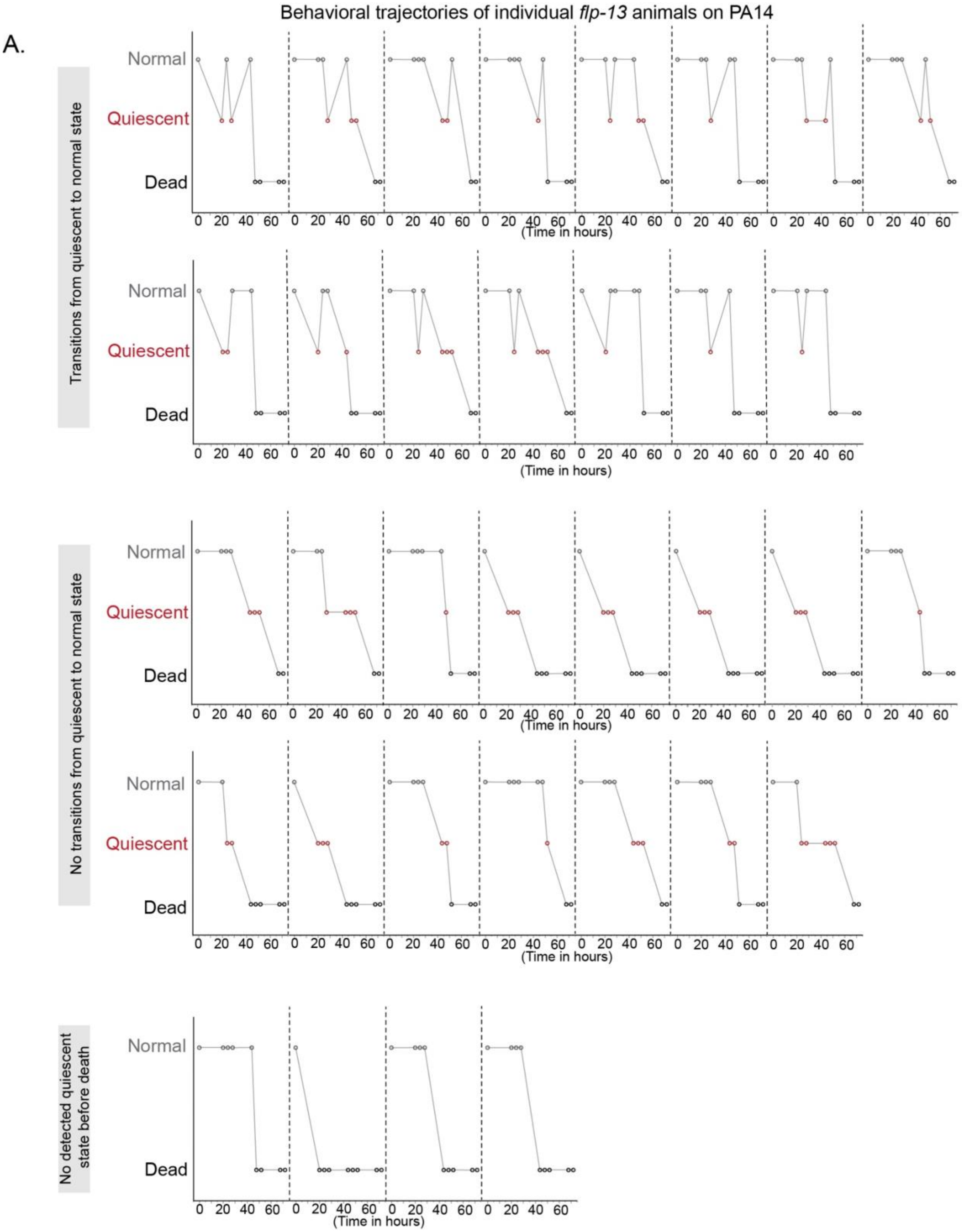

Figure S6, Related to Figure 3

(A) *flp-13* animals were placed individually on PA14 plates and followed from time of addition to death. They were observed at specific timepoints where they were scored as either normal behavior, quiescent (no feeding or locomotion over 20 secs of observation) or dead. Qualitative observations of these behavioral trajectories indicate that a fraction of these animals show transitions back and forth between quiescent states and normal behavior before death, while other animals show a progression from normal behavior to quiescence to death.

Source data are provided as a source data file.

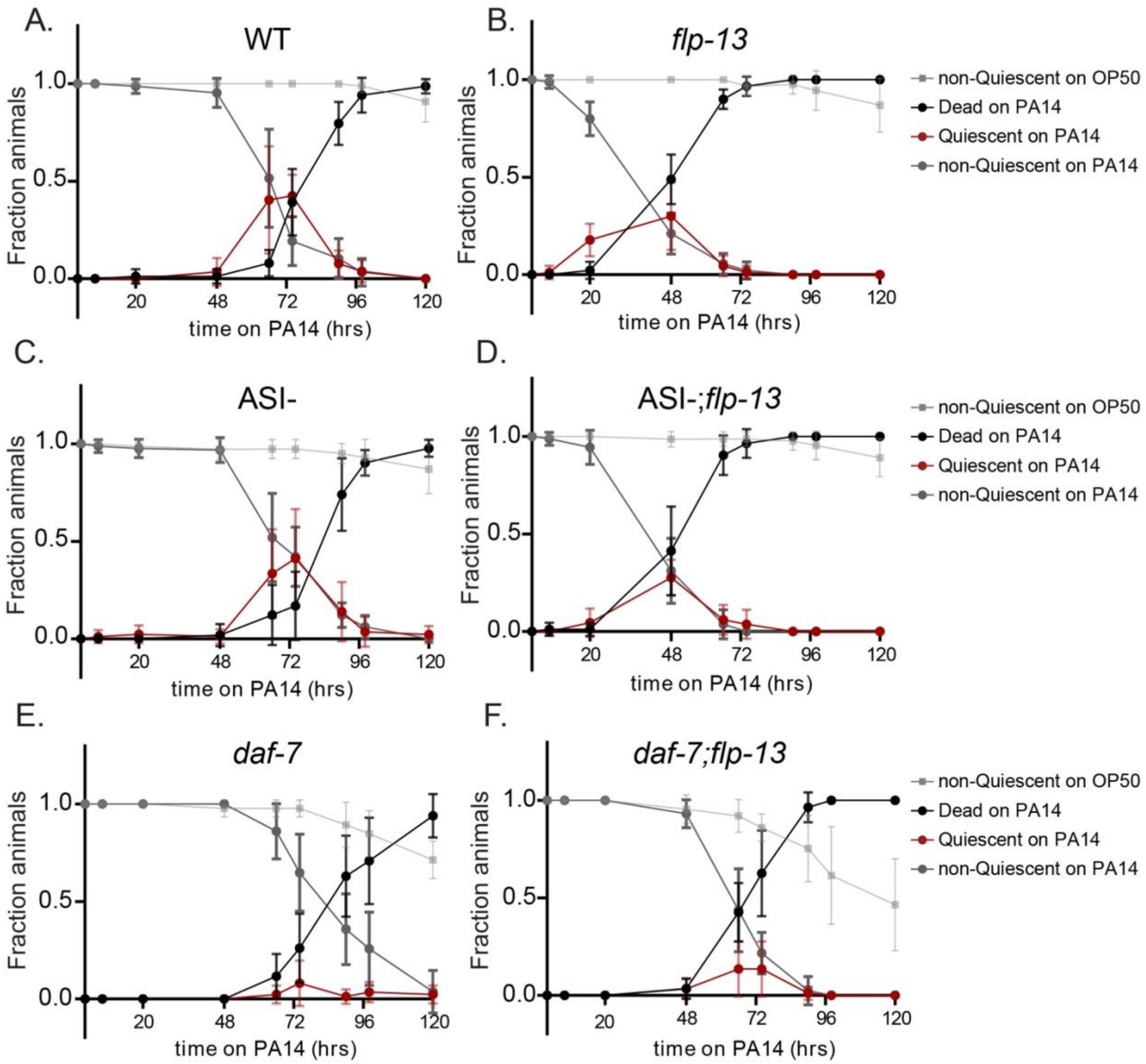

Figure S7, Related to Figure 4

(A-F) Time course analysis of how animal behavior and viability changes over the course of infection. The time indicated on the x-axis is the number of hours since animals were first placed on OP50 or PA14 bacterial lawns as one-day old adults. The line plots depict the fraction of animals that exhibited the indicated phenotypes at that time. Data are shown for wild-type, *flp-13(tm2427)*, (left) ASI::Caspase, *flp-13(tm2427);*ASI::Caspase, (right) *daf-7(e1372ts)* and *flp-13(tm2427);daf-7(e1372ts)* animals. For each genotype, 10 animals were placed on a single plate; three replicates with three plates each were performed.

(A and B) Median time point by which 50% animals died was significantly accelerated in PA14-infected *flp-13* animals, compared to wild-type (WT) (Fig S5A vs S5B). ****p<0.0001, two tailed Mann-Whitney test.

(A, C, E) Median time point by which 50% animals died was not significantly different between PA14-infected WT and ASI- animals (S5A vs S5C,), and between WT and *daf-7* animals (Fig S5A vs S5E). n.s., two tailed Mann-Whitney test.

(B, D, F) Median time point by which 50% animals died was not different between *flp-13* and ASI-;*flp-13* animals (Fig S5B vs S5D), but was significantly delayed in *daf-7; flp-13* compared to *flp-13* animals (Fig S5B vs S5F). **p<0.01., two tailed Mann-Whitney test. Time course for quiescence from these graphs was also represented in Fig 4J.

Source data are provided as a source data file.

SUPPLEMENTARY MOVIE LEGENDS

Supplementary Movie 1. Example 20sec video of a *flp-13* mutant on OP50 bacteria. This is the recording condition used for pumping quantification (these videos were slowed to ¼ speed during playback for quantification, as described in Methods).

Supplementary Movie 2. Example 20sec video of *flp-13* mutant on PA14 bacteria. Data shown as in Supp Video 1.

SUPPLEMENTARY TABLE 1

REAGENT or RESOURCE	SOURCE	IDENTIFIER
Bacterial and Virus Strains		
Escherichia coli: Strain OP50	*Caenorhabditis* Genetics Center	OP50
Escherichia coli: Strain OP50-GFP	*Caenorhabditis* Genetics Center	OP50-GFP
Pseudomonas aeruginosa: Strain PA14	Bob Horvitz Lab	PA14
P. aeruginosa: Strain PA01	Dianne Newman Lab	PA01
Serratia marcescens: Strain Db11	*Caenorhabditis* Genetics Center	Db11
P.aeruginosa: Strain PA14 Δ*lasR*	Lars Dietrich Lab	PA14 *lasR*
P.aeruginosa: Strain PA14 Δ*rhIR*	Dianne Newman Lab	PA14 *rhIR*
P.aeruginosa: Strain PA14 Δ*pqsR*	Dianne Newman Lab	PA14 *pqsR*
P.aeruginosa: Strain PA14 Δ*hcnABC*	Lars Dietrich Lab	PA14 *hcn*
P.aeruginosa: Strain PA14 Δ*phz1/2*	Lars Dietrich Lab	PA14 *phz*
P.aeruginosa: Strain PA14 Δ*rhIA*	Lars Dietrich Lab	PA14 *rhIA*
P.aeruginosa: Strain PA14 Δ*soxR*	Dianne Newman Lab	PA14 *soxR*
Experimental Models: Organisms/Strains		
C. elegans: Wild-type Bristol N2	*Caenorhabditis* Genetics Center (CGC)	N2
C. elegans: *flp-13 (tm2427)*	Sternberg Lab	PS6994
C. elegans: *dmsr-1(qn45)*	CGC	NQ915
C. elegans: flp-13(syb6180 syb6395)	This paper	PHX6395
C. elegans: flvEx431(tag-168p::Cre, elt-2p::GFP) line 4	This paper	SWF1231
C. elegans: flvEx431(tag-168p::Cre, elt-2p::GFP) line 9	This paper	SWF1242
C. elegans: flp-13(syb6180 syb6395); flvEx424(osm-6p::Cre, ceh-34p::Cre, elt-2p::GFP)	This paper	SWF896

C. elegans: flp-13(syb6180 syb6395); flvEx421(eat-4p::Cre, elt-2p::GFP)	This paper	SWF893
C. elegans: flp-13(syb6180 syb6395); flvEx431(tag-168p::Cre, elt-2p::GFP) line 1	This paper	SWF921
C. elegans: flp-13(syb6180 syb6395); flvEx431(tag-168p::Cre, elt-2p::GFP) line 2	This paper	SWF922
C. elegans: flp-13(syb6180 syb6395); flvEx444(flp-24p::Cre, elt-2p::GFP)	This paper	SWF934
C. elegans: flp-13(syb6180 syb6395); flvEx426 (ceh-34p::Cre, elt-2p::GFP)	This paper	SWF943
C. elegans: flp-13(syb6180 syb6395); flvEx426(osm-6p::Cre, elt-2p::GFP)	This paper	SWF944
C. elegans: flp-13(syb6180 syb6395) IV; flvEx465(ceh-53p::Cre, elt-2p::GFP)	This paper	SWF973
C. elegans: flp-13(syb6180 syb6395) IV ; flvEx468(trh-1p::Cre, elt-2p::GFP)	This paper	SWF975
C. elegans: flp-13(syb6180 syb6395) IV; flvEx471(ceh-45p::Cre, elt-2p::GFP)	This paper	SWF979
C. elegans: flp-13(syb6180 syb6395) IV ; flvEx506(ser-2dp::Cre, sra-6p::Cre, elt-2p::GFP)	This paper	SWF1031
C. elegans: flp-13(syb6180 syb6395) IV; flvEx508(tax-4p::Cre, elt-2p::GFP)	This paper	SWF1034
C. elegans: flp-13(syb6180 syb6395) IV; flvEx511(sra-9p::Cre, gcy-5::Cre, gcy-7p::Cre; gcy-33p::Cre, gcy-36p::Cre, elt-2p::GFP)	This paper	SWF1037
C. elegans: flp-13(syb6180 syb6395) IV; flvEx515(ceh-28p::Cre, elt-2p::GFP)	This paper	SWF1041
C. elegans: flp-13(syb6180 syb6395) IV; flvEx566(degt-1p::Cre, elt-2p::GFP)	This paper	SWF1134
C. elegans: flp-13(syb6180 syb6395) IV; flvEx564(sra9p::Cre, gcy-5::Cre, gcy-33p::Cre, gcy-36p::Cre, elt-2p::GFP)	This paper	SWF1132
C. elegans: flp-13(syb6180 syb6395) IV; flvEx565(gcy-7p::Cre, elt-2p::GFP)	This paper	SWF1133
C. elegans: flp-13(syb6180 syb6395) IV; flvEx562(sra-6p::Cre, elt-2p::GFP)	This paper	SWF1130
C. elegans: flp-13(syb6180 syb6395) IV; flvEx563(ser-2d::Cre, elt-2p::GFP)	This paper	SWF1131
C. elegans: dmsr-1(qn45); flvEx460(dmsr-1p(3.5kB)::dmsr1-T2a-GFP; myo2p::mCherry) line 1	This paper	SWF968

C. elegans: dmsr-1(qn45); flvEx460(dmsr-1p(3.5kB)::dmsr1-T2a-GFP; myo2p::mCherry) line 2	This paper	SWF969
C. elegans: dmsr-1 (syb6591) V	This paper	PHX6591
C. elegans: dmsr-1 (syb6591) V; otIs696 [NeuroPAL]	This paper	SWF983
C. elegans: zcIs13 [hsp-6p::GFP + lin-15(+)] V.	CGC	GL347
C. elegans: flp-13(tm2427); zcIs13 (hsp-6p::GFP + lin-15(+))	This paper	SWF995
C. elegans: agIs17 [myo-2p::mCherry + irg-1p::GFP] IV	CGC	AU133
C. elegans: dmsr-1(qn45); agIs17 (myo-2p::mCherry + irg-1p::GFP)	This paper	SWF1052
C. elegans: zcIs4 (hsp-4::GFP)	CGC	SJ4005
C. elegans: flp-13(tm2427); zcIs4 (hsp-4::GFP)	This paper	SWF1114
C. elegans: kyEx4984 (pdfr-1p::BlaC); flp-13(tm2427) IV	This paper	SWF1030
C. elegans: flp-13(tm2427);ceh-17(np1)	This paper	SWF861
C. elegans: flp-13(tm2427); sek-1 (km4)	This paper	SWF982
C. elegans: flp-13(tm2427); nlp-22(gk509904)	This paper	SWF1046
C. elegans: flp-13(tm2427); aptf-1(tm3287)	This paper	SWF1047
C. elegans: flp-13(tm2427); oyls84(gpa-4p::TU#813 + gcy-27p::TU#814 + gcy-27p::GFP +unc-122p::DsRed)	This paper	SWF1053
flp-13; otIs670 [NeuroPAL] V; SWF196-6[MT21793[tag-168::nls-gcamp7f, gcy-28::tagRFP, ceh-36::tagRFP, inx-1::tagRFP, mod-1::tagRFP, tph-1(s)::tagRFP, gcy5::tagRFP, gcy7::tagRFP]] back-crossed 5x to MT21793	This paper	SWF994
C. elegans: flp-13(tm2427); daf-7(e1372ts)	This paper	SWF1055
C. elegans: flp-13(tm2427) IV; flvEx535[srg-47p::GCaMP7f(60 ng/uL), myo3p::mCherry(5 ng/uL)] line 4	This paper	SWF1071
C. elegans: oyls84(gpa-4p::TU#813 + gcy-27p::TU#814 + gcy-27p::GFP +unc-122p::DsRed)	CGC	PY7505
C. elegans: daf-7(e1372ts)	CGC	CB1372
C. elegans: egl-4(n478)	CGC	MT1073
C. elegans: dmsr-1(qn45); egl-4(n478)	This paper	SWF1054
C. elegans: flp-13(tm2427);atfs-1(cmh15)	This paper	SWF880
C. elegans: atfs-1(cmh15)	CGC	SWF879
C. elegans: pmk-1 (km25)	CGC	KU25

C. elegans: tir-1(qd4)	CGC	ZD101
C. elegans: tax-4 (p678)	CGC	CX13078
C. elegans: tbh-1(n3247)	CGC	MT9455
C. elegans: tdc-1(3419)	CGC	MT13113
C. elegans: cat-2(n4547)	CGC	M15620
C. elegans: mod-1(ok103)	CGC	MT9668
C. elegans: nmr-1(ak4)	CGC	VM487
C. elegans: octr-1(ok371)	CGC	CX13079
C. elegans: olrn-1(ut305)	CGC	JC2209
C. elegans: trx-1(jh127)	CGC	KJ912
C. elegans: aex-5(sa23)	CGC	JT23
C. elegans: npr-1(ky13)	CGC	CX4148
C. elegans: ins-11(tm1053)	CGC	FX1053
C. elegans: daf-2(m41)	CGC	DR1564
C. elegans: daf-16(mu86)	CGC	CF1038
C. elegans: aptf-1(tm3287)	CGC	HBR232
C. elegans: ceh-17(np1)	CGC	IB16
C. elegans: ceh-17(tm250)	*C. elegans* National BioResource Project	FX00250
C. elegans: ceh-14(ot900)	CGC	OH15422
C. elegans: flp-7(ok2625) BX2	CGC	SWF1135
C. elegans: flp-24(gk3109)	Paul Sternberg Lab	PS6814
C. elegans: nlp-8(ok1799)	Paul Sternberg Lab	PS6911
C. elegans: nlp-8(ok1799);flp-24(gk3109)	Paul Sternberg Lab	PS6991
C. elegans: flp-13(tm2427);flp-24(gk3109);nlp-8(ok1799)	Paul Sternberg Lab	PS6992
C. elegans: flp-13(tm2427);nlp-8(ok1799)	Paul Sternberg Lab	PS6993

C. elegans: flp-13(tm2427);flp-24(gk3109)	Paul Sternberg Lab	PS6994
C. elegans: flp-13(tm2427);flp-7(ok2625)	Paul Sternberg Lab	PS7084
C. elegans: phm-2(ad597) I	CGC	DA597
C. elegans: tnt-3(ok1011) X	CGC	RB1061
C. elegans: flr-1(ut11) X	CGC	JC55
C. elegans: pbo-1(sa7) III	CGC	JT7
C. elegans: aex-2(sa3) X	CGC	JT3
Chemical Reagents		
Serotonin hydrochloride	Sigma-Aldrich	H9523
5-Fluoro-2′-deoxyuridine (floxuridine, FUDR)	Sigma-Aldrich	F0503
Sodium azide	Sigma-Aldrich	26628-22-8
Software and Algorithms		
MATLAB (2021)	Mathworks	www.mathworks.com
Streampix (v7.0)	Norpix	www.norpix.com
Adobe Illustrator	Adobe	www.adobe.com
NIS Elements	Nikon	www.nikoninstruments.com/products/software
Datavyu	Datavyu	www.datavyu.org
Zeiss Zen 3.7	Zeiss	www.zeiss.com/microscopy/en/products/software/zeiss-zen.html
Fiji (ImageJ)	Schindelin *et al.* (2012)[1]	imagej.net/software/fiji
Other		
SP-20000M-USB3 CMOS camera	JAI	N/A
Micro-NIKKOR 55mm f/2.8 lens	Nikon	N/A
10x25 White Panel LED backlight, 24VDC	Metaphase Technologies	Cat#MS-BL10X25-W-24-ILD-PS
Precision LED Spot Light, 625nm, 40W, Type H	Mightex	Cat#BLS-PLS-0625-030-40-S
BioLED Light Source Control Module	Mightex	Cat#BLS-13000-1

JMP 16	SAS Institute Inc.	www.jmp.com
GraphPad Prism 9.5.1	GraphPad	www.graphpad.com

SUPPLEMENTARY METHODS

Video recording of feeding

Prior to video recordings, we manually scored animals as described above. We used the same plate and bacteria conditions as above and counted grinder movements manually with a tally counter while observing animals for 20 secs. We then video recorded the same plate and animals by mounting a Google Pixel 7a phone on the eyepiece of the microscope (Supp Videos 1-2 for examples). Each animal was video recorded individually for 20 sec at 60 fps. To quantify pumping in these videos, we next slowed down the videos to ¼ speed and counted grinder movements together with time stamps of each grinder contraction using the software Datavyu. Total number of pumps over 20 secs and the time between consecutive full contractions of the grinder were measured. For infected animals, we more precisely examined the very long intervals between grinder movements: these sub-sections of the videos were slowed down further to a speed of $1/8^{th}$, and grinder contraction as well as relaxation were timestamped. Due to the time resolution of the recordings and the latency to click, it was challenging to timestamp the exact time interval between contraction and relaxation, which normally take place within tens of milliseconds. However, the pauses between pharyngeal relaxation and the subsequent pump initiation often extended over hundreds of milliseconds and so could be accurately quantified.

We also tested whether we this reduction in pumping was reflected in reduced intake of fluorescently-tagged bacteria. Animals which were kept on OP50 or PA14 for 20 hours were washed and moved to OP50 tagged with GFP for 1 hour. We did not see a difference in amounts of ingested bacteria in infected animals (Fig S1K). However, this may be attributed to the very low levels of fluorescence observed in both conditions using this assay.

OP50-GFP imaging

One day-old adult animals were placed on OP50-GFP-seeded plates for 20 hours, then picked to a glass petri dish filled with 5ml M9 buffer for 2 minutes before being immobilized on an agar pad with 100uM sodium azide (Sigma Aldrich). *flp-13* mutants and day-matched wild-type controls were tested alongside one another, along with grinder-defective mutants (*tnt-3* and *phm-2*) as positive controls. Images were analyzed using Fiji where the pharynx area was measured from the tip of the nose to just posterior to the grinder, foregut was measured from the region just posterior to the grinder to the anterior end of the gut. Similar regions of interest were drawn around the midbody and tail region of the animal, with similar areas being measured across animals. Mean fluorescence intensities were calculated from these regions and background subtracted.

For checking if feeding reduction could be measured by ingestion of GFP-tagged bacteria, young adult WT animals were kept on OP50 or PA14 for 20 hours as described above. Animals were then added to equivalently seeded OP50-GFP plates for 1 hour, washed in M9 for 5 mins to remove bacteria from the cuticle, and then imaged as described above. Regions of interest were drawn on Fiji and quantified as described above.

Egg-laying assay

10 animals were added to plates seeded as above. Number of eggs on the plate were measured after 1 hour of exposure (0-1 hour). For the 20-21 hour quantification, animals were transferred to an equivalently seeded plate and number of eggs laid after 1 hour was quantified.

For FUDR treatment, 6 cm NGM plates were made with 0.1mg/ml floxuridine (FUDR, Sigma Aldrich), stored at 4°C, and used within 3 days. Plates with or without FUDR were seeded with 100ul OP50 overnight, and L4 animals were placed on them. The next day, animals without any egg development (an expected consequence of FUDR treatment) were selected and placed on a seeded OP50 plate without FUDR for 1 hour, after which pumping rates were quantified manually.

Defection measurement

Five animals were added on plates seeded similarly as above. For each animal, observation started after the onset of defecation and the inter-defecation interval was measured for 5 consecutive defecation events for each animal[2].

Lawn leaving assays

10ul lawns of OP50 or PA14 were seeded on 10 cm low peptone plates for 20 hours at 25°C. Fresh overnight cultures of bacteria were used. 10 animals were added per plate, taking care to not disturb the bacterial lawn during transfer. Animals were recorded on Streampix software at 1 frame every 30 minutes for 20.5 hours using JAI SP-20000M-USB3 CMOS cameras (41mm, 5120x3840, Mono) with Nikon Micro-NIKKOR 55mm f/2.8 lenses. Backlighting was provided by a white panel LED (Metaphase Technologies Inc. White Metastandard 10" X 25," 24VDC). Number of animals on the lawn were measured at each 30 minute interval manually from the recorded images.

Lifespan assay

Lifespan of animals were measured on non-infectious OP50 bacteria[3]. 30 L4 animals were added to individual 6cm plates seeded with 150ul freshly cultured OP50 2 days prior to the start of the experiment. Each strain and bacterial condition were tested with three plates and over three independent replicates. No FUDR was used. Animals were counted as alive or dead every 24 hours until the last animal was marked as dead for WT and *flp-13* strains. Animals were transferred every two days onto freshly seeded plates for the first half of the experiment, until no new progeny were hatching and the lawn was not getting depleted. *daf-2* animals were run as a control in parallel, and the indeed showed longer lifespans. Animals that crawled off the plate edges or died due to picking-related injuries were censored. Animals were considered dead if the animal did not respond to a gentle touch of the pick near its head. Plates were kept in a 22ºC humidified incubator over the course of the experiment.

Latency to reversal

Young adult animals were infected on PA14 or kept on OP50 for 20 hours as described above. A Leica Fluocombi III stereomicroscope was used with a 5X objective to observe onset of reversals. Blue light intensity was kept at 50%, which corresponds to 0.5 mW/cm^2. We titrated the light intensity to a low level that reliably induced reversals in uninfected WT control animals but did

not elicit strong acceleration. We then used this low light intensity to test all other conditions. Due to assay conditions being set up in this way, we only quantified the reliable reversal response. A stopwatch was started coincident with switching on the blue light and time required for onset of a reversal was measured. An inherent latency of about a second by the experimenter handling the two buttons is likely. Only quiescent infected animals and quiescent heat shocked (heat shock was performed as described above) were tested. If animals were right next to each other on the plate and were exposed to similar levels of blue light, only one was tested. A period of 30 seconds was given between testing multiple animals on the plate. Animals were tested without the light sources on to account for any disturbance due to flicking on just the mechanical switch. However, no quiescent animals were roused in the absence of blue light. This confirms that mechanical disturbances did not account for the elevated reversal rates and that instead they are due to blue light stimulation.

SUPPLEMENTARY REFERENCES

1. Schindelin, J. et al. Fiji - an Open Source platform for biological image analysis. *Nat Methods* **9**, 676–682 (2012).

2. Dal Santo, P., Logan, M. A., Chisholm, A. D. & Jorgensen, E. M. The inositol trisphosphate receptor regulates a 50-second behavioral rhythm in C. elegans. *Cell* **98**, 757–767 (1999).

3. Sutphin, G. L., Kaeberlein, M. Measuring Caenorhabditis elegans Life Span on Solid Media. *J. Vis. Exp.* **27**: e1152 (2009).
