# Supplementary material for: Pathogen infection induces sickness behaviors through neuromodulators linked to stress and satiety in C. elegans
Source: Nat Commun. 2025 Apr 3;16:3200. doi: 10.1038/s41467-025-58478-y (PMC11968842; doi:10.1038/s41467-025-58478-y)
Supplement: Supplementary file 2 — Description of Additional Supplementary Files [file 41467_2025_58478_MOESM2_ESM.pdf]

Description of Additional Supplementary Files

Supplementary Movie 1. Example 20sec video of a flp-13 mutant on OP50 bacteria. This is the recording condition used for pumping quantification (these videos were slowed to ¼ speed during playback for quantification, as described in Methods).

Supplementary Movie 2. Example 20sec video of flp-13 mutant on PA14 bacteria. Data shown as in Supp Movie 1.
