# Supplementary material for: Pathogen infection induces sickness behaviors through neuromodulators linked to stress and satiety in C. elegans
Source: Nat Commun. 2025 Apr 3;16:3200. doi: 10.1038/s41467-025-58478-y (PMC11968842; doi:10.1038/s41467-025-58478-y)
Supplement: Supplementary file 5 — Reporting Summary [file 41467_2025_58478_MOESM5_ESM.pdf]

nature portfolio

Corresponding author(s): Steven Flavell

Last updated by author(s): Mar 9, 2025

Reporting Summary

Nature Portfolio wishes to improve the reproducibility of the work that we publish. This form provides structure for consistency and transparency in reporting. For further information on Nature Portfolio policies, see our Editorial Policies and the Editorial Policy Checklist.

Statistics

For all statistical analyses, confirm that the following items are present in the figure legend, table legend, main text, or Methods section.

n/a	Confirmed	
☐	☒	The exact sample size (*n*) for each experimental group/condition, given as a discrete number and unit of measurement
☐	☒	A statement on whether measurements were taken from distinct samples or whether the same sample was measured repeatedly
☐	☒	The statistical test(s) used AND whether they are one- or two-sided<br>*Only common tests should be described solely by name; describe more complex techniques in the Methods section.*
☐	☒	A description of all covariates tested
☐	☒	A description of any assumptions or corrections, such as tests of normality and adjustment for multiple comparisons
☐	☒	A full description of the statistical parameters including central tendency (e.g. means) or other basic estimates (e.g. regression coefficient) AND variation (e.g. standard deviation) or associated estimates of uncertainty (e.g. confidence intervals)
☐	☒	For null hypothesis testing, the test statistic (e.g. *F*, *t*, *r*) with confidence intervals, effect sizes, degrees of freedom and *P* value noted<br>*Give P values as exact values whenever suitable.*
☒	☐	For Bayesian analysis, information on the choice of priors and Markov chain Monte Carlo settings
☒	☐	For hierarchical and complex designs, identification of the appropriate level for tests and full reporting of outcomes
☒	☐	Estimates of effect sizes (e.g. Cohen's *d*, Pearson's *r*), indicating how they were calculated

Our web collection on statistics for biologists contains articles on many of the points above.

Software and code

Policy information about availability of computer code

Data collection	Custom code to process whole-brain imaging data is available on Github (https://github.com/flavell-lab/SparseClustering.jl/releases/tag/v1.0.0 and https://github.com/flavell-lab/euler_gpu/releases/tag/v0.1.1) and Zenodo (DOI: 10.5281/zenodo.14991619 and DOI: 10.5281/zenodo.14991578).
Data analysis	All brain-wide imaging data can be accessed currently using code available on the Readme file on the shared Dryad repository.

For manuscripts utilizing custom algorithms or software that are central to the research but not yet described in published literature, software must be made available to editors and reviewers. We strongly encourage code deposition in a community repository (e.g. GitHub). See the Nature Portfolio guidelines for submitting code & software for further information.

Data

Policy information about availability of data

All manuscripts must include a data availability statement. This statement should provide the following information, where applicable:

- Accession codes, unique identifiers, or web links for publicly available datasets
- A description of any restrictions on data availability
- For clinical datasets or third party data, please ensure that the statement adheres to our policy

Brain-wide imaging data is available on Dryad (DOI: 10.5061/dryad.nvx0k6f2t). In addition, source data has been provided for this paper as an accompanying source data file.

Research involving human participants, their data, or biological material

Policy information about studies with human participants or human data. See also policy information about sex, gender (identity/presentation), and sexual orientation and race, ethnicity and racism.

Reporting on sex and gender	N/A
Reporting on race, ethnicity, or other socially relevant groupings	N/A
Population characteristics	N/A
Recruitment	N/A
Ethics oversight	N/A

Note that full information on the approval of the study protocol must also be provided in the manuscript.

Field-specific reporting

Please select the one below that is the best fit for your research. If you are not sure, read the appropriate sections before making your selection.

☒ Life sciences ☐ Behavioural & social sciences ☐ Ecological, evolutionary & environmental sciences

For a reference copy of the document with all sections, see nature.com/documents/nr-reporting-summary-flat.pdf

Life sciences study design

All studies must disclose on these points even when the disclosure is negative.

Sample size	Sample sizes were determined based on previous literature and norms in the field. All behavior experiments were conducted on at least two different days with multiple experimental replicates.
Data exclusions	For lifespan assays, animals which left the plate and died on the walls were excluded. For quiescence or pumping assays, animals off the bacterial lawn were excluded, since being off food changes feeding rates. A few video recordings that were too blurry to analyze grinder movement were excluded for feeding quantification.
Replication	All experiments were replicated multiple times, as indicated in the figure legends.
Randomization	Animals were allocated to experimental groups randomly and scoring was done in a randomized order.
Blinding	While some early initial pilot experiments were performed unblinded, the vast majority of pumping quantification was performed blinded to genotype and all key experiments were re-confirmed under blinded quantification. Since this did not comprise a sufficient reason for data exclusion, all data is included in the paper. All key behavior experiments were tested by two independent experimenters.

Reporting for specific materials, systems and methods

We require information from authors about some types of materials, experimental systems and methods used in many studies. Here, indicate whether each material, system or method listed is relevant to your study. If you are not sure if a list item applies to your research, read the appropriate section before selecting a response.

Materials & experimental systems

n/a	Involved in the study	
☒	☐	Antibodies
☒	☐	Eukaryotic cell lines
☒	☐	Palaeontology and archaeology
☐	☒	Animals and other organisms
☒	☐	Clinical data
☒	☐	Dual use research of concern
☒	☐	Plants

Methods

n/a	Involved in the study	
☒	☐	ChIP-seq
☒	☐	Flow cytometry
☒	☐	MRI-based neuroimaging

Animals and other research organisms

Policy information about studies involving animals; ARRIVE guidelines recommended for reporting animal research, and Sex and Gender in Research

Laboratory animals	C. elegans
Wild animals	n/a
Reporting on sex	Only C. elegans hermaphrodites were used in this study.
Field-collected samples	n/a
Ethics oversight	n/a

Note that full information on the approval of the study protocol must also be provided in the manuscript.

Plants

Seed stocks	N/A
Novel plant genotypes	N/A
Authentication	N/A
